# Intraductal Papilloma: Atypical Presentation

**DOI:** 10.1155/2013/652728

**Published:** 2013-05-23

**Authors:** P. Ramaswamy, Tanya Khaitan, A. Anuradha, B. Praveen Kumar, S. Sudhakar

**Affiliations:** ^1^Department of Oral Medicine and Radiology, St. Joseph Dental College and Hospital, Duggirala, Eluru, Andhra Pradesh 534003, India; ^2^Department of Oral and Maxillofacial Pathology, St. Joseph Dental College and Hospital, Duggirala, Eluru, Andhra Pradesh 534003, India

## Abstract

Ductal papillomas have unique papillary features arising from the salivary gland duct system. They comprise three rare benign adenomas, namely, inverted ductal papilloma, sialadenoma papilliferum, and intraductal papilloma. Intraductal papilloma is an extremely rare benign salivary gland tumor that occurs most commonly in the minor salivary glands. Here, we are presenting a case report of intraductal papilloma in an 18-year-old patient.

## 1. Introduction

Salivary gland neoplasms are uncommon; only 10–15% of cases are reported in the minor salivary glands [1].

Benign papillary lesions that arise from the ductal system of salivary glands include intraductal papilloma, inverted ductal papilloma, and sialadenoma papilliferum. These ductal papillomas are considered to be relatively rare but generate considerable interest because of their remarkable similarities and variations in clinical and histological appearance [2]. They represent adenomas with unique papillary features, a nonaggressive biologic behavior, and a predilection for the minor salivary glands [3].

Intraductal papilloma is a rare benign lesion showing a characteristic papillary growth of ductal epithelium. It appears as a submucosal small nodule predominantly in lip and buccal mucosa [4]. They tend to occur in the middle-aged and elderly and rarely in children [3]. Here, we are reporting an infrequent case of intraductal papilloma in an 18 year old patient.

## 2. Case Report

An 18-year-old male patient reported having a complaint of growth in the left half of the lower lip region since 2 months. The patient's history revealed that he had noticed the growth 2 months back which initially started as a small growth and gradually increased over a period of 1 month to attain the present size. There was history of mild and occasional pain associated with the growth. The patient claimed that there was no history of paraesthesia or numbness and no similar lesion elsewhere. The medical, dental, family, and personal histories were noncontributory.

**Figure 1:**
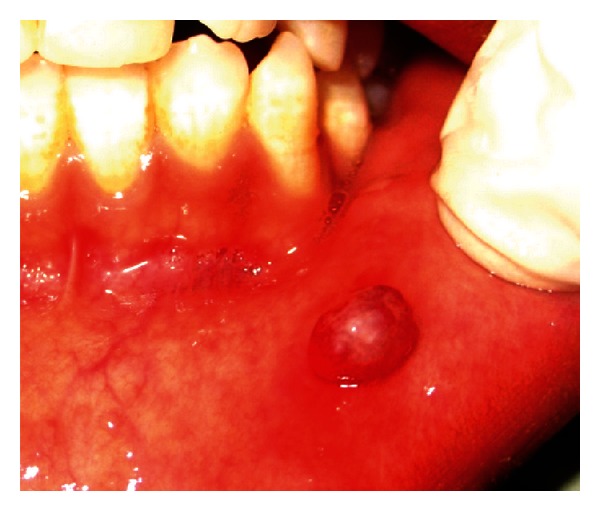
Solitary, well-defined and oval-shaped, nodular exophytic growth on the left half of the lower labial mucosa.

Intraoral examination revealed presence of solitary, well-defined and oval-shaped, nodular exophytic growth (Figure 1) on the left half of the lower labial mucosa measuring approximately 9 × 7 mm  in size. Anteroposteriorly, it extends 0.5 cm from the vermilion border of lower lip to 1 cm from the lower vestibular area. Mediolaterally, the growth extends 1.5 cm away from the midline to 1.5 cm in front of the left corner of mouth. The surface over the growth appears whitish in the centre and erythematous in the periphery. On palpation, the growth was sessile, slight tender, and firm in consistency and arose from the underlying soft tissue.

Based on the history, clinical features and the nature of the growth, a provisional diagnosis of irritational fibroma was considered. Differential diagnoses of fibrosed mucocele, peripheral giant cell granuloma, and extragingival pyogenic granuloma were considered. The patient was subjected to complete hematological examination, and all the parameters were within normal limits. The growth was being excised and specimen was sent for histopathological evaluation.

**Figure 2:**
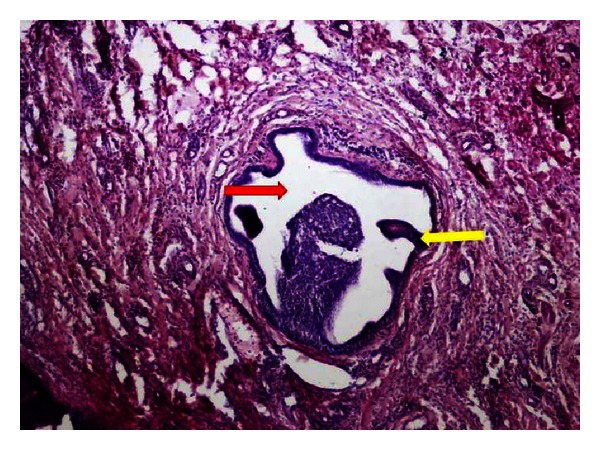
Histopathological section of H&E specimen (40x) showing minor salivary gland with atrophy of the acinar cells (red arrow) and with large dilated duct is partly double walled and partly stratified squamous with numerous papillary fronds (yellow arrow) extending into the lumen.

On microscopic examination, the specimen showed stratified squamous epithelium with acanthosis, parakeratosis, and intracellular edema. The underlying connective tissue is delicate with mild chronic inflammatory infiltrate, numerous dilated engorged capillaries, extravasated red blood cells and mast cells. Minor salivary gland with atrophy of the acinar cells, (red arrow) and with large dilated duct is partly double walled and partly stratified squamous with numerous papillary fronds (yellow arrow) extending into the lumen (Figure 2). The papillary fronds are bland with mucous cells. Based on the clinical features and histopathology, final diagnosis of intraductal papilloma was established.

**Figure 3:**
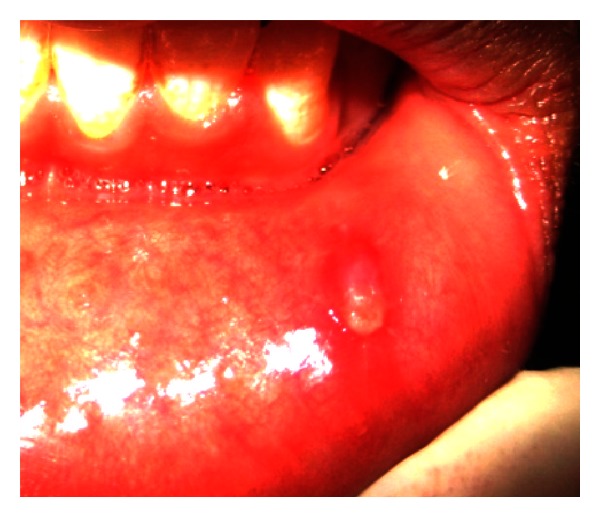
Healing areas seen post-operatively after 10 days.

The patient was being followed up after 10, days and healing lesion was found on examination (Figure 3).

## 3. Discussion

Intraductal papillomas of the breast are relatively common lesions with an incidence of approximately 2–3% in humans [5]. The exact incidence of intraoral intraductal papilloma is not easy to evaluate in the literature due to the inconsistent terminology as the term “papillary cystadenoma” is often applied instead of the term “intraductal papilloma” [2].

Intraductal papilloma is a luminal papillary proliferation of duct epithelium that arises from a segment of the interlobular or excretory duct and causes unicystic dilatation [3]. This is also known as “simple ductal papilloma” [6].

The pathogenesis of intraductal papilloma is still uncertain. Brannon et al. [2] stated that these lesions originate from the salivary gland duct epithelium, most likely the excretory duct.

These tumors usually present as asymptomatic, well-defined solitary submucosal masses or swellings that vary in size from less than 1–1.5 cm [7]. The lesion often has a reddish color [6]. The ages of patients have ranged from 29 to 77 years, with mean age of 54 years. Gender distribution has remained essentially even [7]. The minor salivary glands are more frequently involved than the major glands. Intraductal papillomas are most commonly found in the lips and buccal mucosa followed by palate and tongue. Of the major glands, the parotid is most frequently involved [3].

In contrast to intraductal papillomas, papillary cystadenomas are morphologically multicystic with numerous small-to-medium-sized cystic spaces. In the papillary cystadenoma, the intraluminal growth is often characterized by multiple papillary projections with a variety of epithelial cell types, but usually the papillary growth occupies the lumen to a limited degree [3]. In inverted ductal papilloma, the character of the proliferating epithelium is predominantly epidermoid, whereas in intraductal papilloma, it is columnar or cuboidal epithelium and/or mucous cells [7].

Microscopically, this papilloma appears to arise in the duct system at a deeper level relative to the mucosal surface. The proliferating epithelium is a characteristic of the ductal epithelium in this location. The cyst wall is lined by a single or double layer of cuboidal and columnar cells. Extending into the lumen are several to numerous papillary fronds that have thin fibrovascular cores which form a complex branching structure that creates an appearance of islands of tumor. These papillary projections are covered by the same cuboidal and columnar ductal epithelia [7].

The blockage of a salivary gland duct can result in ductal ectasia and hyperplasia of the ductal epithelium. This epithelial hyperplasia forms the intraluminal papillary projections, and these changes cause chronic inflammation, fibrosis, and atrophy of adjacent salivary gland lobules [2, 4].

Immunohistochemical studies have supported an origin of these tumors in ductal luminal cells, ductal epithelium, and excretory duct epithelium. Ishikawa et al. [8] showed that the tumor cells facing the lumina were strongly positive for keratin and epithelial membrane antigen (EMA) and partly reactive for lactoferrin and S-100. Hara et al. [9] proved that tumor cells show high reactivity to human cytokeratin M-630 and CAM, moderate reactivity to low molecular weight keratin, and partial reactivity to S-100, amylase, and EMA. These reports proposed the excretory duct epithelium as an origin of the lesion. Nagao et al. [10] affirmed that tumor cells are reactive for cytokeratin (AE1/AE3, CAM5.2), EMA, and S-100 and negative for cytokeratin 14, lactoferrin, and SMA and proposed then origin as ductal luminal cells.

Excision is curative, and these tumors are not known to recur. In fact, if small enough, all types of papillomas of the major salivary glands may be removed in the dental clinic with only local anaesthesia [7].
